# The Role of Anxiety, Coping Strategies, and Emotional Intelligence on General Perceived Self-Efficacy in University Students

**DOI:** 10.3389/fpsyg.2019.01689

**Published:** 2019-08-07

**Authors:** Francisco Manuel Morales-Rodríguez, José Manuel Pérez-Mármol

**Affiliations:** ^1^Department of Educational and Developmental Psychology, Faculty of Psychology, University of Granada, Granada, Spain; ^2^Department of Physiotherapy, Faculty of Health Sciences, University of Granada, Granada, Spain; ^3^Instituto de Investigación Biosanitaria ibs.GRANADA, Granada, Spain

**Keywords:** academic performance, emotional intelligence, self-efficacy, university students, coping strategies

## Abstract

The main objective of the present research is to analyze the relationship of levels of self-efficacy and anxiety, coping strategies, and emotional intelligence in Spanish university students. This study has a cross-sectional design. The sample was composed of 258 university students recruited from three academic areas. Descriptive, bivariate, and multivariate regression analyses were performed. Significant bivariate analysis showed a significant inverse correlation between self-efficacy and state anxiety (*r* = −0.340) and trait anxiety (*r* = −0.466). In addition, a direct correlation was found between self-efficacy and the coping strategies of problem-solving (*r* = 0.312), emotional expression (*r* = 0.133), cognitive restructuring (*r* = 0.195), social withdrawal (*r* = 0.103), and coping with a situation (*r* = 0.303), as well as with the emotional intelligence dimensions of emotional clarity (*r* = 0.397) and repair mood (*r* = 0.347). Multivariate regression analysis showed that trait anxiety, problem-solving, emotional expression, social withdrawal, and emotional clarity were significantly related to the dependent variable, predicting 39% of total variance on levels of general perceived self-efficacy. In conclusion, this paper contributes to a better understanding of the related factors to general perceived self-efficacy in undergraduate students.

## Introduction

Self-efficacy was initially defined as the judgments that individuals make about abilities, based on which they organize and execute their actions, in order to achieve the desired performance (Bandura, 1987). General self-efficacy may have significant effects on the behaviors or activities involving the individual, on the effort invested, and on the individual’s thoughts and emotional reactions (Blanco, 2010). At the university context, the general self-efficacy is involved in the judgments that each student makes about his or her abilities to organize and execute the actions required by different specific situations (Sanjuán et al., 2000). The interrelationships between self-efficacy and other psychological and behavioral constructs derive from cognitive-social (clinical) models and psychoeducational approaches. According to the Bandura’s model, with lower overall perceived self-efficacy we may find higher levels of psychological distress such as anxiety and avoidance behaviors (Bandura, 1987; Karademas and Kalantzi-Azizi, 2004; Cabanach et al., 2010). In addition, when certain situations or events exceed the individual’s own capacities, general self-efficacy decreases, and this may play an important role in stress generation. In these situations, coping strategies are needed to manage the high levels of stress (Piergiovanni and Depaula, 2018). Lazarus and Folkman (1984), in its model of coping with stress, conceptualized stress as the interaction between the person and his/her environment. These authors consider stress to be a stimulus–response relationship, where the subject labels a given situation as a threatening or overflowing one that endangers his/her well-being. In turn, stress may lead to alterations in physiological and psychological health (Zajacova et al., 2005).

On the other hand, the psychoeducational approach may be considered as an integral framework for the evaluation and development of constructs such as self-efficacy, coping strategies, anxiety, and emotional intelligence (Organisation for Economic Cooperation and Development (OECD), 2005; Castañeda Fernández, 2016; Morales, 2016; Belaunzaran, 2018). Particularly, the framework for the development of standards for high-quality professional education in psychology – EuroPsy justifies the need to include assessment and intervention competencies related to emotional management, problem-solving, anxiety and interpersonal problems in the higher education setting (Belaunzaran, 2018). In addition, the EuroPsy framework is in line with the guidelines contemplated by the European Higher Education Area (Castañeda Fernández, 2016) and the Organization for Economic Cooperation and Development (Organisation for Economic Cooperation and Development (OECD), 2005), which highlight the importance of developing systemic competencies. Some of these competencies include personal resources, adequate levels of self-efficacy, and low frustration tolerance (Organisation for Economic Cooperation and Development (OECD), 2005; Castañeda Fernández, 2016). Therefore, self-efficacy, the emotional resources of individuals, and coping strategies should be integrated and studied as a whole.

Human emotions may be understood as an emotional continuum that includes anxiety at the negative pole and emotional intelligence at the positive pole (Cropanzano et al., 2003). Commonly, anxiety may be divided into two clinical dimensions: state anxiety, referring to how one feels at the moment, and trait anxiety, representing how one generally feels. Castillo et al. (2016) showed high levels of state anxiety, trait anxiety, and academic stress in university students. For this population, many research studies have shown that there may be several stressors that cause high level of anxiety such as examinations, lack of time to undertake academic activities, and academic overload. For this reason, several authors highlight that variables such as individual expectations and perceived self-efficacy should be considered to cope with or cushion the effects of stress and anxiety in the higher education setting (Shankland et al., 2010; Hendy et al., 2014; Ruiz, 2014; Mathews et al., 2016). People who doubt their abilities may believe that things are more difficult than they really are (Pajares and Schunk, 2001; Contreras et al., 2005). In fact, some investigations indicate that self-efficacy is associated negatively with anxiety (Luszczynska et al., 2005; Olivari Medina and Urra Medina, 2007; Bueno-Pacheco et al., 2017).

As for emotional intelligence in the educational context, a recent study has shown the importance of the so-called intrapsychic factors, such as the management of emotions in the effective coping with stress (Cabanach et al., 2016). Students with low or medium levels of emotional clarity (individual’s meta-knowledge of their affective experience) seem to perceive the environment as more threatening and show greater psychophysiological responses to stress than those with high emotional clarity. Furthermore, students with low emotional attention (ability to detect threatening information quickly) value beliefs about the performance and the value of content in a more stressful way than students with high emotional attention (Boden et al., 2013). In the same vein, a research analyzed the differences in the perception of academic stressors and psychophysiological responses of stress based on different profiles of emotional regulation. Students with higher levels of acceptance and control over their emotions perceived the circumstances or the academic environment as less threatening, and therefore, suffered lower levels of psychological stress (González-Cabanach et al., 2017). Different studies also point to the importance of certain socio-emotional factors in learning, such as organizational, personal, and social obstacles, underlining the need to pay more attention to them. These obstacles direct attention toward self-efficacy and its related factors in university students (Salanova et al., 2010; Medrano et al., 2016; Del Rosal and Bermejo, 2017).

With regard to coping strategies, these are defined as efforts to regulate emotions, behaviors, cognitions, psychophysiology, and environmental aspects in response to the stress of everyday events. Each problem or situation requires the use of a specific coping strategy. Therefore, the same strategy can be effective or ineffective depending on whether or not the individual perceives the situation as threatening (Lazarus and Folkman, 1984; Carver et al., 1989). More optimistic people use more effective strategies; however, in uncontrollable situations they tend to use strategies considered ineffective such as acceptance or resignation to the problem (Scheier et al., 1986). Higher education students may face many stressful changes and transitions that usually increase the number of stressors that students face in this field. Denovan and Macaskill (2013) found that university students apply different types of coping strategies such as optimism, hope, and self-control in stressful situations to facilitate the adjustment and adaptation process. The choice of one coping strategy or another may be determined by the level of self-efficacy perceived (Vandercleyen et al., 2014). Different studies have found an association between the perception of self-efficacy and the coping strategies employed (Vandercleyen et al., 2014; Zambianchi and Ricci-Bitti, 2014; Gárriz et al., 2015; Piergiovanni and Depaula, 2018); however, these have not included emotional continuum of individuals such as anxiety (in a positive sense) or emotional intelligence (in a negative sense) in the same model.

Many research studies have shown the importance of general and academic self-efficacy in the educational context. Self-efficacy has been classically defined as perceived capabilities within specific domains (Bandura, 1987; Pajares, 1996; Schunk and Pajares, 2002). Academic self-efficacy is understood as the “students’ perception of their own ability to achieve the proposed activity, in the process of which students interpret the results of their activities and academic tasks” (Piergiovanni and Depaula, 2018, p. 18). Students may differ in their beliefs about their learning skills or if they adapt “effectively” to the context of learning (Schunk, 1991; Piergiovanni and Depaula, 2018). Both constructs, general and academic self-efficacy, appear to have an influence on academic performance and the development of adaptive academic goals (Pajares, 1996; Valentine et al., 2004; Wolf et al., 2018). However, although academic self-efficacy is an important aspect in the educational field, general self-efficacy can give a more global view of the students’ perception of themselves in several stressful contexts of everyday life (Sanjuán et al., 2000). That is, although self-efficacy may be projected as a specific domain factor, a higher or lower self-efficacy can be also interpreted in a general manner to identify the global trust or the generalized judgment when students face novel or stressful situations (Luszczynska et al., 2005; Bueno-Pacheco et al., 2017). With this background, we might expect some generality of self-efficacy from educational domain to other contexts (Schunk and Pajares, 2002). Therefore, the study of general self-efficacy and related factors is becoming increasingly relevant in the educational context, since this information could be used to achieve academic success, an effective approach to the educational process, and an improvement in students’ quality of life.

However, as far as we know, there are no studies focused on analyzing the relationship between levels of general self-efficacy and psychoeducational and/or clinical variables, such as strategies for coping with everyday stress and the emotional dimension, whether positive (emotional intelligence) or negative (anxiety) in the university population. Knowledge of these relationships would help to improve the learning process, providing guidance, vocational, counseling, and support services with strategies that will enhance the students’ management of emotions and the coping with certain conflict situations of daily living. This information could also promote interventions that increase the general perception of self-efficacy, prevent stress in the educational setting, and lead to better academic performance. For these reasons, the main objectives of this study are (1) to evaluate the levels of general self-efficacy, anxiety, coping strategies, and emotional intelligence in a sample of university students; (2) to analyze if anxiety, coping strategies, and emotional intelligence are related to the levels of general self-efficacy in this population.

## Materials and Methods

### Research Hypotheses

The research hypotheses of the current study are: (1) a sample of the Spanish university students reports high levels of general self-efficacy and anxiety, and low levels of coping strategies and emotional intelligence; (2) the general self-efficacy is related to anxiety, coping strategies, and emotional intelligence in the higher education context; i.e., the higher levels of general self-efficacy, the lower the levels of anxiety, the higher the performance in coping stress, and the higher the emotional intelligence.

### Design

This is an observational-descriptive study.

### Participants

**Figure 1:**
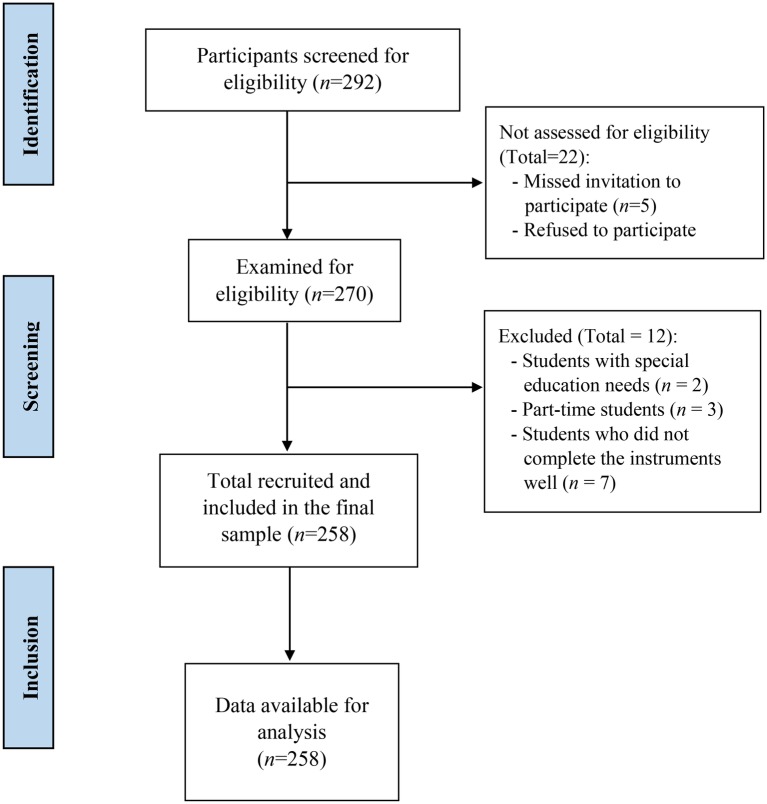
Flow diagram of subjects who participated in the study according STROBE statement.

The global sample was initially composed of 270 university students recruited from three academic areas of the University of Granada, Granada, Spain. The data were collected from January to November 2017. After applying the selection criteria, the final sample was composed of 258 students. In the final sample, 94 (36.4%) are men and 164 (63.6%) women. The mean age of the participants is 21.5 years (SD = 3.7); and they are aged between 18 and 45 years. The sample belongs to the academic area of humanities, social and health sciences. There were no differences between academic areas for sex and age. A flow chart, with the participant selection process, is depicted in Figure 1, following the STROBE guidelines for observational studies (Von Elm et al., 2014).

The inclusion criteria established for this study were: (1) being a full-time university student and (2) aged between 18 and 65 years old. The exclusion criteria were: (1) students with special education needs (disabilities or severe behavioral disorders). Participants were informed about the objectives and procedures of this study, and it was conducted according to the Declaration of Helsinki. All participants gave their written informed consent before being included in the recruitment phase. The present study protocol was approved by the Ethics Committee of the University of Granada (Granada, Spain), with registration number: 328/CEIH/2017. The participants completed an individual informed consent to participate in the study.

### Procedures

Undergraduate students were approached at the conclusion of a lecture during semester one of 2018 by a non-teaching member. They were asked to complete a hard copy of the questionnaires included in the study. The participants were informed that collected data would remain anonymous and used only for research purposes. It took participants on average 40 min to complete self-report questionnaires. The participants placed the completed questionnaires in the back of the lecture theater as they exited.

### Instruments

Sociodemographic data were collected by a self-elaborated questionnaire. It includes information such as age, sex, academic area, academic year level, and academic performance (with a score ranging from 0 to 10 points). The academic performance was calculated by the mean of scores obtained in all subjects passed until the study completion, i.e., the mean value of the average grade of the student record.

#### General Self-Efficacy Scale

The questionnaire has 10 items based on the Likert scale ranging from 1 to 10, where 1 is never, and 10 is always (Baessler and Schwarzer, 1996; Sanjuán et al., 2000). This unidimensional scale assesses general self-efficacy in coping with stress. This scale has a Cronbach’s alpha of 0.87 and a high validity index and has been used in numerous studies beforehand (Sanjuán et al., 2000; Serra, 2010; Espada et al., 2012). The present study has shown a reliability index for this scale of a Cronbach’s alpha of 0.91.

#### State Trait Anxiety Inventory

It is made up of a total of 40 items, where the first 20 measure state anxiety (STAI-state anxiety scale) or how one feels at the moment, and the last 20 measure trait anxiety (STAI-trait anxiety scale) or how one generally feels (Spielberger et al., 1970). The items present specific conditions; respondents are asked to indicate the level of anxiety experienced in each condition, rating it on a Likert scale from 0 (=nothing) to 3 (=a lot). This inventory has a Cronbach’s alpha of 0.90 for state anxiety and 0.94 for trait anxiety (Buela-Casal et al., 1982). In the current study, the state trait anxiety Inventory (STAI) has shown a Cronbach’s alpha of 0.85.

#### Coping Strategies Inventory

This test is divided into two parts (Tobin et al., 1989). In the first part, the participants are asked to think about a problem that worries them and externalize it. In the second part of the test, participants are asked to answer 40 questions considering the problem; they have to rate their responses on a 4-point Likert scale, where 0 = absolutely nothing and 4 = completely. These 40 items are grouped into subscales: problem-solving, self-criticism, expressing emotions, wishful thinking, social support, cognitive restructuring, problem avoidance, and social withdrawal. This instrument has been used on many occasions due to its high reliability and validity (Cano et al., 2007). In the current study, the Coping Strategies Inventory has shown a Cronbach’s alpha ranging from 0.73 to 0.87.

#### Trait Meta-Mood Scale – TMMS-24

The Trait Meta-Mood Scale measures different dimensions of emotional intelligence (Salovey et al., 1995). This scale includes items whose answers are rated on a Likert scale that ranges from 1 to 5, where 1 indicates that the person does not agree at all and 5 that he/she fully agrees. It is made up of a total of 24 items that measure three subscales: the attention that each person pays to his/her own feelings, that is, if they are able to feel and express feelings properly (items 1–8); the clarity with which emotional states are understood (items 9–16); and, finally, the ability to regulate emotional states correctly, called repair (items 17–24). This instrument is highly reliable, attaining a Cronbach’s alpha of 0.90 for attention, 0.90 for clarity, and 0.86 for repair (Fernández-Berrocal et al., 2003). In the present study, this instrument has shown a Cronbach’s alpha ranging from 0.77 to 0.82.

### Data Analysis

SPSS software (version 20.0) was used for statistical analysis. First, descriptive analysis was performed and normal distribution of variables was confirmed with the Kolmogorov–Smirnov test. To determine the association between demographic variables, levels of anxiety, coping strategies, and emotional intelligence and self-efficacy scores, the Pearson correlation coefficient, and a multiple regression model were used. General self-efficacy was determined as the dependent variable, and age, state anxiety (STAI-state anxiety scale), trait anxiety (STAI-trait anxiety scale), coping strategies (Coping Strategies Inventory subscales of problem-solving, self-criticism, emotional expression, wishful thinking, social support, cognitive restructuring, problem avoidance, social withdrawal, coping with a situation), and emotional intelligence (TMMS-24 subscales of emotional attention, clarity, and repair) as independent variables. Multiple regression analysis included only those independent variables significantly correlated to self-efficacy following a stepwise multiple regression model. A *p* of less than 0.05 was regarded as a statistically significant value in all cases.

### Sample Size

In a previous study (Domínguez-Lara, 2016), a significant bivariate correlation of 0.217 between academic self-efficacy and coping styles, measured with the EAPESA scale (assessing self-efficacy) and COPEAU scale (evaluating coping styles), was used to calculate the sample size required to detect such effect size in the sample, using G*power 3.1 software. This calculation showed that a sample size of 225 university students was needed to provide a confidence interval of 95%, with a power of 95%, assuming a level of bilateral significance (*α*) of 0.05. To avoid possible missing data, drop out of participants, or badly completed instruments, the recruited sample should be increased by 10%. Hence, the final sample should include at least a minimum of 248 participants.

## Results

### Descriptive Statistics

The sample is composed of 117 undergraduate students (45.3%) from first year; 60 from second year (23.3%); 33 from third year (12.8%); 26 from fourth year (10.1%); and 22 from fifth year (8.5%). Table 1 shows the descriptive statistics of the average score and the different scales of general self-efficacy (General self-efficacy scale), anxiety (STAI-state anxiety scale and STAI-trait anxiety scale), coping strategies (Trait Meta-Mood Scale), and emotional intelligence (TMMS-24).

**Table 1:** *Mean* (SD) and 95% CI of academic performance, state and trait anxiety, self-efficacy, coping strategies, and emotional intelligence (*N* = 258).

	*M*	SD	95% CI	
			Lower bound	Upper bound
Academic performance (0–10 points)	7.81	0.87	7.11	8.64
Perceived self-efficacy	73.74	9.26	72.65	74.92
**Anxiety**				
State anxiety	22.81	9.60	21.72	24.03
Trait anxiety	23.21	9.41	22.12	24.42
**Coping strategies**				
Problem-solving	13.92	4.32	13.41	14.41
Self-criticism	8.71	5.32	8.13	9.44
Emotional expression	10.62	4.91	10.02	11.23
Wishful thinking	12.31	4.92	11.73	12.93
Social support	13.23	4.83	12.63	13.84
Cognitive restructuring	10.41	4.22	9.94	10.92
Problem avoidance	7.01	3.92	6.51	7.41
Social withdrawal	7.02	4.64	6.42	7.52
Coping with a situation	2.73	1.21	2.62	2.82
**Emotional intelligence**				
Emotional attention	26.61	7.01	25.82	27.41
Clarity	25.83	6.62	25.03	26.73
Repair	26.82	6.12	26.01	27.51

*M: mean; SD: standard deviation; Confidence interval: 95%*.

### Relationship Between Perceived Self-Efficacy and Academic Performance, State and Trait Anxiety, Coping Strategies and Emotional Intelligence Components

Bivariate analysis showed a significant inverse correlation between self-efficacy and state anxiety (*r* = −0.340) and trait anxiety (*r* = −0.466). In addition, a direct correlation was found between self-efficacy and the coping strategies of problem-solving (*r* = 0.312), emotional expression (*r* = 0.133), cognitive restructuring (*r* = 0.195), social withdrawal (*r* = 0.103), and coping of situation (*r* = 0.303), as well as with the emotional intelligence dimensions of emotional clarity (*r* = 0.397) and repair mood (*r* = 0.347). Bivariate Pearson correlation between academic performance, anxiety, coping strategies, emotional intelligence, and perceived self-efficacy is shown in Table 2.

**Table 2:** Bivariate Pearson correlation between perceived self-efficacy and other independent variables (academic performance, anxiety, coping strategies, emotional intelligence).

Variables	Self-efficacy	
	Pearson *r*	*p*
Age (years)	0.120	0.055
Academic performance (0–10 points)	0.009	0.899
**Anxiety**		
State anxiety	−0.340^**^	<0.001
Trait anxiety	−0.466^**^	<0.001
**Coping strategies**		
Problem-solving	0.312^**^	<0.001
Self-criticism	−0.057	0.358
**Emotional expression**	0.133^*^	0.033
Wishful thinking	−0.078	0.212
Social support	0.111	0.076
Cognitive restructuring	0.195^*^	0.002
Problem avoidance	0.093	0.137
Social withdrawal	0.103^*^	0.005
Coping with a situation	0.303^**^	<0.001
**Emotional intelligence**		
Emotional attention	0.101	0.106
Clarity	0.397^**^	<0.001
Repair	0.347^**^	<0.001

* *p < 0.05*;

** *p < 0.001*.

Multivariate regression analysis showed that trait anxiety, problem-solving, emotional expression, social withdrawal, and clarity were significantly related to the dependent variable, predicting the 39% of total variance (adjusted *R*^2^ = 0.386, *F* = 25.760, *p* < 0.001) on levels of general perceived self-efficacy, in university students. The variables that emerged as predictors were trait anxiety (*p* < 0.001), problem-solving (*p* < 0.001), emotional expression (*p* = 0.037), social withdrawal (*p* = 0.039), and clarity (*p* < 0.001). There was no collinearity among the included variables in the regression model. Table 3 shows the final multiple regression model of self-efficacy after the selection of independent variables.

**Table 3:** Regression model of anxiety, coping strategies, and emotional intelligence predictive associated factors to perceived self-efficacy in university students.

Perceived self-efficacy (adjusted *r*^2^ = 0.386)						
Independent variables	*B*	95% CI		*β*	*SE*	*p*
		Lower bound	Upper bound			
**Anxiety**						
Trait anxiety	−0.469	−0.596	−0.342	−0.459	0.064	<0.001
**Coping strategies**						
Problem-solving	0.442	0.197	0.686	0.210	0.124	<0.001
Emotional expression	0.247	0.016	0.479	0.126	0.118	0.037
Social withdrawal	0.278	0.014	0.541	0.125	0.134	0.039
**Emotional intelligence**						
Clarity	0.355	0.180	0.530	0.250	0.089	<0.001

*r^2^, regression coefficient of determination; B, regression coefficient; CI, confidence interval; β, adjusted coefficient from multiple linear regression analysis; SE, coefficient standard error*.

### Discussion and Conclusions

The main objective of the present study has been to evaluate if anxiety, coping strategies, and emotional intelligence are related to the levels of self-efficacy in a sample of Spanish university students. The results from the bivariate analyses showed that general perceived self-efficacy is statistically related to state and trait anxiety, the coping strategies of problem-solving abilities, emotional expression, cognitive restructuring, social withdrawal, and coping, in addition to the emotional intelligence aspects of emotional clarity and mood repair. In turn, of all the variance in self-efficacy, 39% was predicted by a model including anxiety, problem-solving, emotional expression, social withdrawal, and emotional clarity. This can be explained because self-efficacy is associated with how university students feel, think, and act in the academic scenario. Poor performance in academic tasks or assignments due to limited personal resources (such as ineffective coping strategies and low emotional intelligence) could be causing a lower level of self-efficacy in these students (Shankland et al., 2010; Bodys-Cupak et al., 2016; Huerta et al., 2017). However, these associations are cross-sectional; therefore, we cannot determine cause and effect. On the other hand, academic performance was not related to self-efficacy. Nevertheless, given that general self-efficacy was measured, it is not appropriate to assume that the participants’ self-efficacy does represent their academic self-efficacy. For this reason, it is notable that academic performance does not correlate with general self-efficacy.

Regarding anxiety, this study showed that state and trait anxiety are factors associated with self-efficacy. In other words, when the anxiety components or symptoms increase, the levels of self-efficacy decrease, or vice versa. These results may be explained because the assignments, classes, tutorial attendance, and exams can be a source of stress that university students have to cope with. Therefore, higher levels of anxiety may be a risk factor for low levels of self-perception of efficacy, and showing high levels of general self-efficacy perception could be a protective factor against anxiety. Nevertheless, only trait anxiety was a significant predictor of self-efficacy, probably because it is more stable and provokes more mental suffering and makes us more vulnerable to our own negative thoughts. It is likely that university students may have developed a negative recurrent thinking process focused on good academic performance since they usually have to foresee and overcome diverse situations to pass the subjects. Several studies have found a similar relationship (Finney and Schraw, 2003; Shankland et al., 2010; Hendy et al., 2014; Domínguez-Lara, 2016). Ruiz (2014) concluded that both the levels of general self-efficacy and those of sensitivity to anxiety are independent predictors of pathological concern. Shankland et al. (2010) reported that high levels of academic self-efficacy and adequate coping styles have a positive influence on anxiety symptoms and adaptation to the educational scenario in students from an alternative teaching context.

Regarding coping strategies, factors such as problem-solving abilities, emotional expression, cognitive restructuring, social withdrawal, and coping were related to self-efficacy in university students, with problem-solving, emotional expression, and social withdrawal being significant predictors of this construct. In line with this, Crego et al. (2016) found that problem-solving, positive re-evaluation, and search for social support have a positive effect on self-efficacy; however, other coping strategies such as venting negative emotions and negative auto-focus have a negative effect. Cabanach et al. (2010) found that higher scores are achieved in a student’s academic self-efficacy if they use active coping styles such as planning and positive re-evaluation. Nevertheless, these authors found no relationship between search for social support and the level of self-efficacy. The investigation performed by Tsarenko and Strizhalova (2013) analyzed the effects of the level of self-efficacy on active, expressive, and denial strategies. The main finding reports a negative effect of self-efficacy on the expressive strategy. Domínguez-Lara (2016) found that students with higher academic self-efficacy rely more on their academic abilities and have a greater tendency to use strategies such as task orientation and preparation. Bodys-Cupak et al. (2016) evaluated the effect of self-efficacy on coping strategies and stress levels in nursing students. They showed that the students who report higher scores on self-efficacy show lower stress levels and use more active coping strategies such as search for social support, planning, positive re-evaluation, and active solutions. Along the same lines, Zhao et al. (2015) found that transference is the coping strategy used more frequently by these students. In addition, this study revealed that self-efficacy is connected with the frequency of use of optimistic strategies and problem-solving (Chan et al., 2009; Zhao et al., 2015).

Regarding emotional intelligence, this study shows a relationship between self-efficacy and emotional clarity and mood repair, emotional clarity being a significant predictor in the perception of efficacy. There is previous evidence on the effect of emotional intelligence on the self-efficacy of professionals in relation to decision-making (Jiang, 2014). However, to our knowledge, there are no studies including this aspect in combination with anxiety and coping strategies to explain self-efficacy. The study performed by Ruiz-Aranda et al. (2013) highlighted that female student health professionals with higher emotional intelligence experience lower perceived stress, and higher levels of satisfaction and happiness. Therefore, training programs of emotional intelligence may help university students to cope with part of the challenges they encounter in health science disciplines as well as increasing general self-efficacy in coping with stress. Adeyemo (2007) stated that emotional intelligence and academic self-efficacy are critical components in academic performance. Moreover, this researcher highlighted that emotional intelligence can be a teachable construct.

In conclusion, the results from the present study have shown that general perceived self-efficacy is related to state and trait anxiety, the coping strategies of problem-solving abilities, emotional expression, cognitive restructuring, social withdrawal, and coping with a situation, as well as the emotional intelligence aspects of emotional clarity and repair mood. Self-efficacy is predicted by trait anxiety, problem-solving, emotional expression, social withdrawal, and emotional clarity, and vice versa.

### Limitations, Educational Implications, and Future Directions

Several limitations are present in this study and should be considered. First, participants took part voluntarily in the study and were not selected randomly. However, it was not considered ethical to obligate that students participate in the study. Secondly, participants may have answered items in a socially desirable form since the general self-efficacy scale and the other instruments are self-report scales therefore, person-positivity bias may have been present. Thirdly, students were recruited from several academic areas but they were located in a single university form a specific geographical location; hence, the generalizability of the findings may be limited. In the fourth place, the cross-sectional design of the study does not allow to establish a causal association between exposure and outcome.

Several educational implications arise of current findings such as they may help to improve the learning process, providing guidance, vocational, counseling and support services with strategies that will enhance the students’ management of emotions and the coping with certain conflict situations. In addition, the results of the study can be used to design interventions that increase the general perception of self-efficacy, prevent stress in the educational setting, and lead to better academic performance.

Hence, future studies should evaluate the effect of educational interventions considering self-efficacy and strategies for coping with everyday stress and the emotional dimension, whether positive (emotional intelligence) or negative (anxiety) in the university population. On the other hand, since the study was unicentric, future research in this topic should be multicentric by including students’ data from several educative centers. Additionally, multi-level analysis could be performed by taking into consideration the specific needs and demands by discipline, geographical location, or sociodemographic characteristics (sex, level of course, or academic performance).

## Ethics Statement

Participants were informed about the objectives and procedures of this study, and it was conducted according to the Declaration of Helsinki. All participants gave their written informed consent before being included in the recruitment phase. The present study protocol was approved by the Ethics Committee of the University of Granada (Granada, Spain), with registration number: 328/CEIH/2017. The participants completed an individual informed consent to participate in the study.

## Author Contributions

FM-R designed the study and contributed to bibliographic review, article writing, and data analysis. JP-M contributed to article writing and data analysis. Both authors revised the article critically and approved the final manuscript.

### Conflict of Interest Statement

The authors declare that the research was conducted in the absence of any commercial or financial relationships that could be construed as a potential conflict of interest.
